# Gene-dosage- and sex-dependent differences in the prodromal-Like phase of the F344tgHD rat model for Huntington disease

**DOI:** 10.3389/fnins.2024.1354977

**Published:** 2024-02-07

**Authors:** Veronika Ratz-Wirsching, Johanna Habermeyer, Sandra Moceri, Julia Harrer, Christoph Schmitz, Stephan von Hörsten

**Affiliations:** ^1^Department of Experimental Therapy, University Hospital Erlangen, Erlangen, Germany; ^2^Preclinical Experimental Center, Friedrich-Alexander-University, Erlangen-Nürnberg, Erlangen, Germany; ^3^Chair of Neuroanatomy, Institute of Anatomy, Faculty of Medicine, Ludwig-Maximilian University of Munich, Munich, Germany

**Keywords:** Huntington disease, behavioral phenotyping, sex differences, gene-dosage, prodrome, striatal atrophy

## Abstract

In Huntington disease (HD) the prodromal phase has been increasingly investigated and is currently in focus for early interventional treatments. Also, the influence of sex on disease progression and severity in patients is under discussion, as a sex-specific impact has been reported in transgenic rodent models for HD. To this end, we have been studying these aspects in Sprague Dawley rats transgenic for HD. Here, we took up on the congenic F344tgHD rat model, expressing a fragmented *Htt* construct with 51 CAG repeats on an inbred F344 rat background and characterized potential sexual dimorphism and gene-dosage effects in rats during the pre-symptomatic phase (1–8 months of age). Our study comprises a longitudinal phenotyping of motor function, emotion and sensorimotor gating, as well as screening of metabolic parameters with classical and automated assays in combination with investigation of molecular HD hallmarks (striatal cell number and volume estimation, appearance of HTT aggregates). Differences between sexes became apparent during middle age, particularly in the motor and sensorimotor domains. Female individuals were generally more active, demonstrated different gait characteristics than males and less anxiolytic-like behavior. Alterations in both the time course and affected behavioral domains varied between male and female F344tgHD rats. First subtle behavioral anomalies were detected in transgenic F344tgHD rats prior to striatal MSN cell loss, revealing a prodromal-like phase in this model. Our findings demonstrate that the congenic F344tgHD rat model shows high face-validity, closely resembling the human disease’s temporal progression, while having a relatively low number of CAG repeats, a slowly progressing pathology with a prodromal-like phase and a comparatively subtle phenotype. By differentiating the sexes regarding HD-related changes and characterizing the prodromal-like phase in this model, these findings provide a foundation for future treatment studies.

## 1 Introduction

Huntington disease (HD) is an autosomal dominant, neurodegenerative disorder, which is genetically characterized by a pathologically expanded CAG repeat sequence (>39) in exon 1 of the *IT15* gene located on chromosome 4 with a strong correlation between the CAG repeat length and the age of disease onset (Snell et al., 1993; The Huntington’s Disease Collaborative Research Group, 1993). The expansion results in an elongated polyQ stretch near the N-terminus of the so-called mutant huntingtin protein (mHTT), which accumulates in neurons and forms aggregates leading to degeneration of the affected cells (DiFiglia et al., 1997; Scherzinger et al., 1997; reviewed in: Bates, 2003). Atrophy is especially present in basal ganglia and the neocortex of HD patients due to predominant loss of striatal GABA-ergic medium spiny neurons (MSN) and cortical pyramidal neurons (Halliday et al., 1998; Waldvogel et al., 2014; Jiang et al., 2023).

Clinically, patients suffer from a triad of slowly progressive symptoms, i.e., often early mood and mental disturbances, followed by a hyperkinetic movement disorder and late cognitive decline that vary in severity and time of onset. First subtle emotional and cognitive deficits are already detectable about 15 years prior to any motor symptoms during the so-called prodrome. Subsequently, the beginning of the symptomatic phase is characterized by minor motor impairments (e.g., clumsiness or unstable gait), emotional disturbances, (e.g., anxiety or depression) and first subtle cognitive difficulties (e.g., problems with time estimation, decision making). Later, motor impairment, particularly choreatic movements, as well as deterioration of gait, speech and swallowing, and further decline of cognitive functions occur. In the final stage of the disease, patients display severe weight loss and chorea transitions into rigidity, bradykinesia and dystonia (Sanberg et al., 1981; Johnson et al., 2014). Despite ongoing efforts, therapy today pursues a mere symptomatic approach and patients usually die within 10–15 years after occurrence of the first motor symptoms (Dickey and la Spada, 2018). Lately, more attention is given to the pre-symptomatic as well as the prodromal phase (Blockx et al., 2012; Siebzehnrübl et al., 2018; Wood, 2018). In the latter, first subtle changes are already detectable but motor impairment and pathological damage are still widely absent. Therefore, this phase is considered ideal for early intervention studies (Jiang et al., 2023). Ongoing preclinical research should focus even more on early onset alterations in an attempt to specify the prodromal-like phase and being of special interest to open the change for early diagnosis and to facilitate therapy of this deadly disease.

Similar to other neurodegenerative diseases, the clinical picture of HD appears to be influenced by the patient’s sex. The majority of studies describe a slower and milder progression with higher prevalence for emotional disturbances in women (Foroud et al., 1999; Pekmezovic et al., 2007; Epping and Paulsen, 2011; Reedeker et al., 2012; McAllister et al., 2021). Contradictory reports, however, stating no influence of sex exist as well (Zielonka et al., 2013; Dale et al., 2016). Size and ethnicity of the study cohorts and the environmental factors taken into account seem to substantially influence whether significant sex differences become evident or not (Pringsheim et al., 2012; Meoni et al., 2020). Based on current literature, it can be said that the role of patients’ sex is controversially discussed but yet poorly understood. It is most probably accountable for some of the variability observed between patients, but more investigation is needed to fully determine the role of sex as biological variable in HD patients.

Animal models, which are normally more standardized and less influenced by environmental factors than human individuals, reflecting HD pathology show clear differences between sexes on molecular and behavioral levels as well, apparently independent of the genetic construct, background or species. Similar to the human situation, a more severe phenotype is seemingly present in male animals (Dorner et al., 2007; Bode et al., 2008; Kuljis et al., 2016; Padovan-Neto et al., 2019). A common misconduct in neuroscientific studies is the omission of sex differentiation or bias toward usage of exclusively male animals, which prevents the possibility to refine studies in the sense of the 3R principle and more importantly leads to impaired translatability, constituting an unacceptable risk for any interventional study (Shansky and Woolley, 2016; Will et al., 2017).

The F344/HanSvh-Tg (tmHTT51CAG) (F344tgHD) strain is one of the very few rat models for HD (reviewed: Carreira et al., 2013). It constitutes a backcrossed version of the thoroughly characterized SPRDtgHD model (von Hörsten et al., 2003) with a truncated *Htt* construct comprising 51 CAG repeat. Homozygous males have been shown to reflect major key symptoms of human HD pathology like striatal volume reduction at 21 months and an early behavioral phenotype (Plank et al., 2018). A comprehensive sex comparison of this model as well as in-depth analysis of potential gene-dosage (zygosity) effects and advanced investigations into motor deficits as well as early, prodromal-like symptoms are still owing (Plank et al., 2018). Classical motor tasks as performed by Plank et al. (2018) so far failed to detect eventual early motor impairments. Therefore, this study presents a comprehensive, longitudinal phenotyping with classical as well as automated behavioral assays in combination with investigation of molecular HD hallmarks (striatal MSN loss, striatal volume reduction, presence of HTT aggregates) in female and male F344tgHD rats aged 1–8 months. Our study describes a prodromal-like phase in F344tgHD rats, where subtle behavioral impairments are already detectable while significant striatal cell loss and volume reduction is still absent. Additionally, we observed clear sexual dimorphism especially in the motor and sensorimotor domains in all three genotypes, stressing the need to distinguish between sexes to refine future studies and thereby increase the translatability.

## 2 Materials and methods

### 2.1 Animals

#### 2.1.1 Generation and genotyping of the congenic F344tgHD rat line

Generation of the F344tgHD rat line has been described in detail (Plank et al., 2018). Briefly, this line derives from the Sprague Dawley HD rat model (von Hörsten et al., 2003) and represents a truncated HD model with expression of 727 amino acids (equaling 22%) of the full length *Htt* gene under the endogenous rat *Htt* promotor. Breeding of the experimental cohort was done by het × het mating to exclude genetical bias by strict maternal/paternal inheritance or any potential impact of genotype differences in nursing quality. Genotyping was performed with probe-based real time PCR (Plank et al., 2018).

#### 2.1.2 Experimental subjects

For this study, we compared heterozygous (het) and homozygous (hom) F344tgHD rats of both sexes with age-matched, wildtype (wt) counterparts. Females used for this study were excluded from breeding to avoid any potential effects of maternity on the investigated parameters.

Rats were housed in sex- and age-matched, stable social groups of maximum four individuals per cage with all three genotypes present. Food (Ssniff lab chow pellets, Germany) and ozonized tap water were available *ad libitum* under 12 h dark: light cycle.All animal procedures and experiments were conducted according to local and ARRIVE guidelines (Kilkenny et al., 2010; Percie du Sert et al., 2020) and approved by the ethical boards of the District Government of Lower Franconia, Bavaria, Germany (Approval number: 55.2-2532-2-1349).

### 2.2 Study design

**FIGURE 1:**
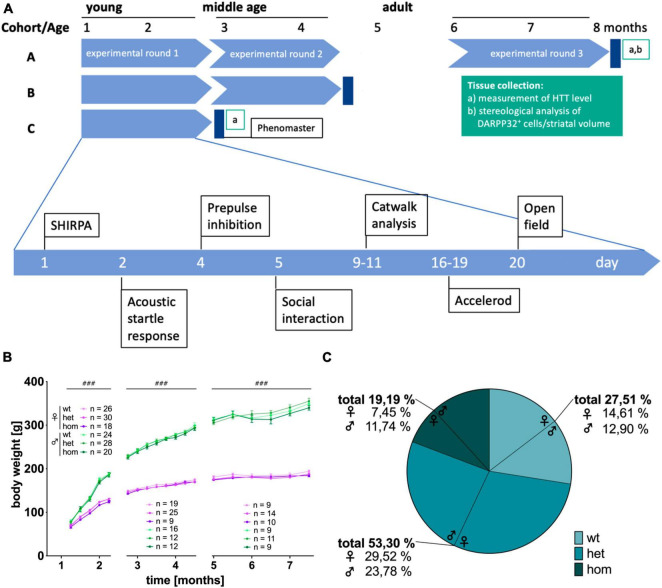
Study design, body weight and mendelian distribution of breeding in the F344tgHD rat model. **(A)** Study design of the longitudinal (factor “age”), cross sectional (factor “sex” and “genotype”) behavioral characterization of F344tgHD rats. **(B)** Body weight development during experimental phase. No genotype effect could be detected but a clear sex-dependent influence on body weight was visible from 5 weeks of age on. Data is shown as mean ± SEM. Hashtags indicate significant statistical effects between sexes (^###^*p* < 0.001). **(C)** The mendelian distribution of wildtype, heterozygous and homozygous offspring was calculated in percentage for 53 breeding events resulting in 349 living pups.

The study was designed to screen for early sex- and genotype-dependent differences in the congenic F344tgHD rat model for Huntington’s disease. Three experimental cohorts (A-C) of male and female, wt, het and hom animals of the F344tgHD rat model were investigated using a test battery covering motor and emotional as well as ethological behaviors and metabolism monitored within an automated home cage-like test system (PhenoMaster System, TSE) (Figure 1A). Three Cohorts were investigated: Cohort A was tested at young (1–3 Months), middle (3–5 Months), and adult age (5–8 Months). Cohort B was tested at young and middle age and Cohort C only at young age. Each Cohort was investigated with the home cage-like Phenomaster system after the last experimental round and then sacrificed for brain extraction. The order of behavioral experiments per test phase was strictly defined and the exact age of the animals at each experiment follows the order within each test phase. The exact number of animals used per test varies due to technical reasons and is summarized in the supplements (Supplementary Table 1) or can be found in the respective graph in the results section. Brains were collected after the last experimental trial at an age of 3, 5, and 8 months and investigated with immunohistochemistry as well as stereological and molecular biological approaches (*n* = 5 per group, unless stated otherwise).

All behavioral experiments were conducted during the light phase of the light: dark cycle (7 to 2 h prior to the dark phase). The automated home cage phenotyping lasted 72 h comprising three full light: dark cycles.

### 2.3 Behavioral assessment

Male and female animals were tested on the same day using the same equipment to minimize variance among animals. To prevent olfactory disturbances, equipment was cleaned with ethanol after every animal.

#### 2.3.1 General health

All animals were screened for general health following a published protocol (Urbach et al., 2010) comprising parameters like morphology, sensory function and neurological reflexes at the beginning of the first experimental test. No animals had to be excluded prior to or during the experimental periods due to health issues. Body weight was measured as additional indicator for normal development and health and was conducted once a week until 5 months and once every 2 weeks thereafter.

#### 2.3.2 Accelerod

Motor coordination was investigated with the accelerating RotaRod system for rats (TSE Systems GmbH, Germany) as described previously (Plank et al., 2018). The number of training trials was 3 per day for 3 consecutive days. The performance of the animals was measured as “time spent on RotaRod” while the turning rate increased from 4 rpm to maximum 40 rpm with a maximum total time of 5 min. Due to their small size, young rats and middle-aged females did not fall from the RotaRod with increasing speed but hold on to the wheel, rotating with it. To include this observation in the evaluation of the experiment, turning around with the wheel more than one time was also considered as “fall down.”

#### 2.3.3 Catwalk

Quantitative assessment of motor performance was performed using Catwalk XT System (Noldus Information Technology, Wageningen, Netherlands). Each rat performed the CatWalk test on two consecutive days within each test phase, where it had to complete 10 runs in maximum two rounds / day. All runs were carried out in the dark with no external stimuli. Walks were analyzed with the CatWalk software 10.6.608. Runs were evaluated manually and defined as compliant whenever the variation of velocity within one run was smaller than 60% and the animal crossed the whole pathway without pausing, sniffing, turning or rearing. Furthermore, the toe spread and print length were extrapolated, where appropriate, and measured manually within the software. CatWalk systems allows investigation of over 250 parameters. In this study, only the most relevant parameters for HD were further examined and included, i.e., base of support (BOS, average width between either front or hind paws), swing speed (speed of the paw during swing phase, where paw is not in contact with glass plate), stride length (distance between successive placement of the same paw) and velocity. Secondary parameters that were investigated included coefficients of variants defining intraindividual gait variability and the regulatory index, reflecting the number of normal step sequence patterns in relation to the total number of paw placements. In line with a previously published work by our group (Timotius et al., 2019), parameters influenced by body length (stride length, swing speed, velocity, BOS) were scaled accordingly to correct for potential bias caused by potential age-, sex- or genotype-related size differences.

#### 2.3.4 Open field test and opisthotonos-like head movements

Open field test (OF) was conducted as described (Plank et al., 2018). Briefly, the conflict between novelty-induced exploratory behavior and the animal’s innate aversion to an unfamiliar open arena is triggered and can be analyzed for various motoric and anxiety-related parameters (Gould et al., 2009). In this setup, the observation time was prolonged to 15 min. Analyzed parameters comprised time spent in center of the arena (CenT), grooming and time spent rearing. All parameters were detected with Ethovision software v8 (Noldus Information Technology, Wageningen, Netherlands) and analyzed automatically, except for grooming and rearing, which were analyzed manually. Opisthotonos-like head movements defined as abrupt, rapid, brief and unsustained irregular movements of the neck (Cao et al., 2006) were evaluated quantitatively and a representative video displaying those changes can be found in the supplements.

#### 2.3.5 Social interaction test of anxiety

The social interaction test (SI) of anxiety, where two rats, unfamiliar to each other are placed in an arena, was performed according to a published protocol (Urbach et al., 2014; Plank et al., 2018) with 5 min of observation. Here, an internal conflict between social interaction with an unfamiliar, non-related partner and anxiety to a novel surrounding is intentionally triggered. Rats of the experimental cohort, that were equal in sex, genotype and age, non-co-housed, non-littermate, and unfamiliar to each other were tested in pairs of two. Active social interaction was manually scored as the time spent by the pair of rats sniffing and following each other.

#### 2.3.6 Acoustic startle response and pre-pulse inhibition of startle

Basal sensorimotor coupling was examined via measurement of the animal’s response to an acoustic stimulus (acoustic startle response, ASR) and the inhibition of this reaction through a weaker pre-stimulus (pre-pulse inhibition of startle, PPI).

Both experiments were conducted as previously described with a 4-place startle response system (TSE Systems GmbH, Germany) (Urbach et al., 2014; Plank et al., 2018; Habermeyer et al., 2020).

#### 2.3.7 Automated behavioral and metabolic phenotyping

Circadian patterns of locomotor activity, ingestion and calorimetry were conducted in a home cage-like environment with a modular, 12-place PhenoMaster System (TSE Systems GmbH, Germany) as previously described (Urbach et al., 2014; Habermeyer et al., 2020) . For this purpose, animals were single-housed for a standard period of 72 h under visual and audio contact. Data were collected and analyzed with the TSE PhenoMaster Software, V4.4.6.

### 2.4 Biochemical analysis

#### 2.4.1 Tissue collection and sample preparation

Tissue was collected as described in Habermeyer et al. (2020). Briefly, animals were anesthetized, transcardially perfused with ice-cold perfusion buffer [0.01 M phosphate buffer (pH 7.4), 0.8 % sodium chloride, 0.024 % potassium chloride, 0.05 % sodium bicarbonate] and brains were removed. One hemisphere was fixed in 4% PFA for 2 h, equilibrated in 30 % sucrose and cut into 40 μm coronal sections for immunohistology. The other half of the brain was dissected into individual regions and snap-frozen with −76°C isopentane for molecular biological analysis. For Western Blot analysis, snap-frozen tissue was homogenized with NPER buffer (Thermo Scientific, Cat: 87792) and protease inhibitor (cOmplete™ mini protease inhibitor cocktail, Merck, Cat: 11836153001) using a pebble mill (46 Hz, 45 s) and subsequent UV sonication (10 s, 40%). Homogenates were centrifuged (20800 × *g*, 4°C, 30 min) and protein levels were measured via Bradford Assay (Roti^®^-Quant, Roth, Cat: K015).

#### 2.4.2 Immunohistological analysis

Coronal brain sections were washed in PBS, and PBS + 0.2 % TritonX-100 (30 min) to remove remains of the storing solution. An antigen retrieval step with 10 min 0.25 % glutardialdehyde treatment, followed by washing steps in PBS and again 10 min in 95% formic acid was performed prior to a 30 min peroxidase block in PBS containing 0.3 % H_2_O_2_, followed by washing steps in PBS-T. Unspecific antibody binding was blocked with 5 % normal donkey serum (Jackson ImmunoResearch Labs Cat# 017-000-001, RRID:AB_2337254). Mouse anti-polyQ antibody (Millipore Cat# MAB1574, RRID:AB_94263) was used as primary antibody, diluted 1:30000 and incubated for 16 h at 4°C. After washing off unbound primary antibody, biotin labeled donkey-anti-mouse antibody was applied 1:500 for 1 h. After washing with PBS, ABC-reagent (Vector Laboratories, Burlingame, CA, USA, #PK-6100) was applied for 30 min, followed by a washing step. Staining was developed with the DAB peroxidase substrate kit (Vector Laboratories, Burlingame, CA, USA, #SK-4100) with Nickel chloride solution as color enhancer according to manufacturers’ specifications for 4 min followed by washing in tap water and PBS. Immunofluorescence stained sections were washed in PBS-T, incubated with DNA-marker 4′,6-Diamidino-2-phenylindole (DAPI, Thermo Fisher Scientific Cat# D3571, RRID:AB_2307445, 1:10000) for 3 min and washed with PBS-T. All sections were mounted on glass slides (Thermo Fisher Scientific, Waltham, MA, USA). Immunohistochemistry-slides were dried overnight, dehydrated in ascending ethanol solutions and xylol, before adding DPX mounting medium (Sigma-Aldrich, St. Louis, MO, USA) and cover slip. Immunofluorescence-slides were shortly dried and cover-slipped with Mowiol-488.

Sections were analyzed on a Keyence BZX800 microscope and images were taken with the corresponding BZX800 imaging and sectioning software modules (Keyence Corp., Osaka, Japan). Post-processing was performed in Adobe photoshop CC (version 2015.1.2; Adobe systems, San José, CA, USA).

#### 2.4.3 Stereological analysis

Female and male hom, het and control (wt) F344tgHD rats were used at 8 months of age (*n* = 3, respectively). Brains were removed and processed as described above. Every sixths section (40 μm) including the striatum was stained with anti-DARPP32 antibody (Abcam Cat# ab40801, RRID:AB_731843) for 72 h at 4°C, with two interstitial 8 h periods at room temperature. Anatomical boundaries of the striatum were adapted from Kántor et al. (2006). Z-stacks (1 mm steps) were taken with an Olympus BX51-DSU microscope and an UPLSAPO objective 20x (numerical aperture: 0.75). Unbiased analysis of the number of DARPP32^+^ cells was conducted by an experimenter, blinded to the animals’ genotype and sex with the optical fractionator method/Stereo Investigator^®^ Software (MicroBrightField, Williston, VT, USA). The height of the counting sites was set to 10 μm with a 3.5 μm guard zone above and below. Grid size was determined as 500 μm × 500 μm with a systematic random sampling and frame size as either 150 μm × 150 μm or 100 μm × 100 μm, depending on the cell density. On average, 311 counting frames were counted manually per animal. Striatal volume was evaluated using the Cavalieri principle/Stereo Investigator^®^ Software (MicroBrightField, Williston, VT, USA) with 100 μm grid spacing and a randomized grid rotation.

#### 2.4.4 Protein analysis

Protein level of mutant and physiological HTT were measured via Western Blot analysis with mouse-anti-HTT antibody (Millipore Cat# MAB2166, RRID:AB_2123255, 1:1000) on 7,5% stain free tris-glycine gels (BioRad, Cat# 4568021). A total of 50 μg of whole protein lysates were separated by SDS-PAGE, activated with UV light and electroblotted onto PVDF membranes (Immun-Blot LF PVDF, Bio-Rad, Cat: 1620264). As loading control and for later correction for small differences regarding the total amount of protein loaded, a picture of the total protein was taken before blocking unspecific binding sites with 5 % BSA (Serva, Cat: 11930.03) in TBS-T (0,1 % Tween20) for 2 h at room temperature. The primary antibody was incubated over night at 4 °C in blocking solution and detected with a horseradish peroxidase-conjugated secondary antibody (rabbit-anti-mouse IgG, Sigma, Cat: A9044; goat-anti-rabbit IgG, Sigma, Cat: A9169; 1: 3000 each). Signals were visualized with enhanced chemiluminescence (Immobilon^®^ Forte, Millipore, Cat: WBLUF0100). All blots were analyzed with the Image Lab™ software (version 6, Bio- Rad) following the Quick Start Guide for Western Blot Normalization (BioRad, Bulletin 6434, 2015). Levels of the respective protein were normalized to total protein levels of the corresponding sample (intra-sample normalization) with the same wild type control serving as reference lane on every blot (inter-blot normalization). During the normalization process, a normalization factor is calculated, using the following formula:


$$n\,o\,r\,m\,a\,l\,i\,z\,a\,t\,i\,o\,n\,f\,a\,c\,t\,o\,r=\frac{\begin{array}{cc} t\,o\,t\,a\,l\,v\,o\,l\,u\,m\,e\,\left(i\,n\,t\,e\,n\,s\,i\,t\,y\right)\,o\,f & \\ s\,t\,a\,i\,n-f\,r\,e\,e\,r\,e\,f\,e\,r\,e\,n\,c\,e\,l\,a\,n\,e \end{array}}{\begin{array}{cc} t\,o\,t\,a\,l\,l\,a\,n\,e\,s\,t\,a\,i\,n-f\,r\,e\,e\,v\,o\,l\,u\,m\,e & \\ \left(i\,n\,t\,e\,n\,s\,i\,t\,y\right)\,o\,f\,e\,a\,c\,h\,l\,a\,n\,e \end{array}}$$

The normalized volume depicted in the figure is than calculated by multiplying volume (intensity) of each protein band with the normalization factor.

All samples were analyzed in three individual experiments and averaged for further statistical analysis.

### 2.5 Statistical evaluation

Data was analyzed with a 3-way analysis of variance (ANOVA) (factor “genotype,” “sex,” “age”) to verify an age effect. Afterward, data was split and differences in the mean values between groups were compared within each age-point using a 2-way ANOVA with two between-subject factors (“genotype” and “sex”). Either Tukey’s (factor: genotype) or Šídák (factor: sex) correction was used for *post hoc* multiple comparison.

Statistical analysis of body weight was performed using a 3-way ANOVA for repeated measures (factor “time”) with two between-subject factors “genotype” and “sex.” Statistical analysis was conducted separately for time periods 6–8, 9–18, 20–30 weeks of age to correct for different group sizes due to sample collection. A total of 2-way ANOVAs were performed with Prism9.3 (GraphPad Software, San Diego, CA, USA). A total of 3-way ANOVAs were performed with Jamovi open software (v2.3.18).

For all analysis, a statistically significant difference between groups was defined as *p < 0.05, **p < 0.02, ***p < 0.001, and ****p < 0.0001. Genotype-differences are indicated by asterisks, sex-differences by hashtags and interaction by paragraphs. Further statistical information comprises F_(*DFn, DFd*)_. All data are presented as mean ± SEM.

## 3 Results

This study aimed at characterizing early behavioral and molecular alteration in transgenic F344tgHD animals with special emphasis on potential sex-dimorphism (see Figure 1A for study design). It comprises a longitudinal behavioral analysis as well as stereological and molecular investigation of HD hallmarks. All investigated parameters showed a significant age-effect not further indicated in the graphs for clearer illustration.

### 3.1 F344tgHD\rats are of general health but show deviation of mendelian ratio in genotypes

Longitudinal observation of F344tgHD rats did not show apparent differences in general health between sexes, nor genotypes. No anomalies, like tremor, staggering gait, seizures (neither spontaneous nor handling-induced), ataxia or obvious tumor developments were observed within the experimental period of 8 months. Adult transgenic females demonstrated opisthotonos-like movements of the head observed during the open field test. Detailed evaluation is described in the corresponding results section. Determination of steroid hormones (17ß-estradiol, progesterone and testosterone) in plasma revealed physiological level for both sexes without any apparent difference between genotypes (Supplementary Figure 1). All animals continuously gained weight over time reaching a stable weight at adulthood independent of their genetic condition (Figure 1B) [5–8 weeks: *F*_(3,580)_ = 929.867, *p* < 0.001; 9–18 weeks: *F*_(7,624)_ = 2182.255, *p* < 0.001; 20–30 weeks: *F*_(5,367)_ = 251.43, *p* < 0.001]. No difference in weight was detected at any age point due to the animals’ genotype. A strong but physiological difference between males and females was present from 5 weeks on [5–8 weeks: *F*_(1,582)_ = 136.522, *p* < 0.001; 9–18 weeks: *F*_(1,630)_ = 1664.423, *p* < 0.001; 20–30 weeks: *F*_(1,372)_ = 1002.193, *p* < 0.001].

Breeding via het × het mating of F344tgHD animals produced litter sizes of 6.98 ± 2.48 pups. A total of 50 breeding events resulted in 349 living descendants of which 27.51 % were wt, 53.30 % het and 19.19 % hom animals (Figure 1C). The genotype ratio in all offspring was 1:1.94:0.68 (wt:het:hom) with a significant difference between sex [*p* = 0.001, *F*_(2, 182)_ = 7.201]. This discrepancy between theoretical (1:2:1) and actual Mendelian ratio manifested especially in female animals, where the ratio was 1:2.02:0.51, while 1:1.84:0.91 in the male litter. A total of 49 pups died before genotyping due to cannibalism or other unknown factors.

### 3.2 Motor phenotype

#### 3.2.1 Female F344tgHD rats demonstrate superior motor skills in the accelerod test independent of their genotype

**FIGURE 2:**
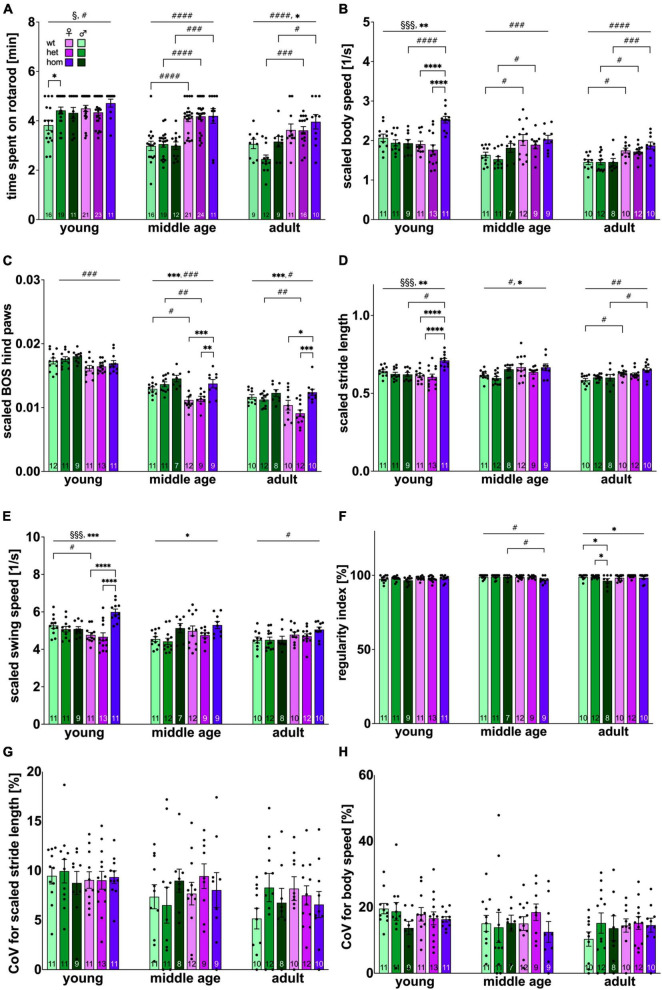
Locomotion performance and coordination of F344tgHD rats. **(A)** Coordination and balance measured with the classical Rotarod system. **(B–H)** Locomotion and gait defining parameters resulting from Catwalk XT system analysis. Selected parameters of high translational value for clinical HD features were plotted. **(B–E)** Were normalized to body size to correct for a potential bias due to sex dimorphism as refined lately (Timotius et al., 2019). Data is presented as mean ± SEM. Hashtags indicate significant statistical effects between sexes (^#^*p* < 0.05, ^##^*p* < 0.02, ^###^*p* < 0.001, ^####^*p* < 0.0001). Asterisks indicate significant genotype differences (*p < 0.05, **p < 0.02, ***p < 0.001, ****p < 0.0001). Paragraphs indicate significant interaction between factors “sex” and “genotype” (^§^*p* < 0.05, ^§§§^ < 0.001). The investigated n is indicated at the bottom of each bar. The legend is depicted in **(A)**.

The animals’ motor coordination and balance were assessed with the classical accelerod paradigm. All rats successfully passed the training period reaching a stable level of performance (Supplementary Figure 2). Final results obtained at each age point are depicted in Figure 2A. Time spent on the accelerod significantly decreased over time [*F*_(2,256)_ = 46.082, *p* < 0.001]. At all ages, females demonstrated significantly better performance than males [young: *F*_(1,95)_ = 5.913, *p* = 0.0169; middle age: *F*_(1,97)_ = 68.96, *p* ≤ 0.0001; adult: *F*_(1,61)_ = 23.75, *p* < 0.0001]. *Post hoc* comparison computed significant sex differences in wt (young: *p* = 0.006; middle age: *p* < 0.0001), het (middle age: *p* < 0.0001; adult: *p* = 0.0001) and hom animals (middle age: *p* = 0.0001; adult: *p* = 0.0422). At young age, a significant sex × genotype interaction [*F*_(2,95)_ = 3.636, *p* = 0.0301] was measured in addition, describing significant better performance of het compared to wt males (*p* = 0.0154). At adult age, a main genotype effect [*F*_(2,61)_ = 3.368, *p* = 0.041] was observed though not yielding significant differences at the level of pairwise comparisons.

#### 3.2.2 F344tgHD rats show subtle genotype-specific gait abnormalities in the CatWalk performance with sex-dependent variance in the affected subdomain

CatWalk test was performed to quantitatively assess gait and locomotion performance of the animals.

Since significant changes in body silhouette length were detected over the investigated time period (Supplementary Figure 3A), parameters influenced by body length were scaled as published recently (Timotius et al., 2019). All *a priori* “translational” and/or “face-valid” parameters (i.e., interesting for comparisons with clinical motor features of HD) (Vandeputte et al., 2010; Abada et al., 2013; Pyo et al., 2017) were plotted and are depicted in Figures 2B–H.

Measurement of velocity (scaled body speed) (Figure 2B) revealed a significant decrease over time [*F*_(2,173)_ = 30.29, *p* < 0.001]. Subsequently, a significant factor interaction [*F*_(2,60)_ = 9.949, *p* = 0.0002] as well as a genotype effect [*F*_(2,60)_ = 7.591, *p* = 0.0012] was present at young age. Significant faster speed was detected for hom females compared to hom males (*p* = 0.0003) as well as heterozygous (*p* < 0.0001) and homozygous (*p* < 0.0001) females. Interestingly, this genotype effect was not persistent in older age, but a significant sex-dependent difference toward faster locomotion in females manifested instead [middle age: *F*_(1,53)_ = 13.78, *p* = 0.0005; adult: *F*_(1,56)_ = 31.51, *p* > 0.0001] with significant group comparison between female and male wt (middle age: *p* = 0.0219; adult: *p* = 0.0160) and het (middle age: *p* = 0.0457; adult: *p* = 0.0174) animals, as well as hom animals (*p* = 0.0008) at adult age.

The base of support (BOS) describes the average width between either front or hind paws. In this analysis, pronounced effects were only found in the hind paws and BOS of front paws can be found in the supplements (Supplementary Figure 3B). BOS significantly decreased over time [*F*_(2,171)_ = 273.632, *p* < 0.001]. Overall BOS was significantly higher in males at all investigated ages [young: *p* = 0.0008, *F*_(1,61)_ = 12.36; middle age: *p* < 0.0001, *F*_(1,53)_ = 17.49; adult: *p* = 0.0102, *F*_(1,56)_ = 7.074] (Figure 2C). *Post hoc* analysis resulted in significant difference between wt (*p* = 0.0173) animals at middle age and between het animals at middle (*p* = 0.0028) and adult age (*p* = 0.0062).

Furthermore, a genotype difference was detected beginning with middle age [*F*_(2,53)_ = 10.26, *p* = 0.0002] and persisting at adulthood [*F*_(2,56)_ = 9.013, *p* = 0.0004] with increased BOS for hom females (middle age: wt vs. hom: *p* = 0.0005, het vs. hom: *p* = 0.0026; adult: wt vs. hom: *p* = 0.0205, het vs. hom *p* < 0.0001).

Measurement of swing speed and stride length did not reveal differences between front and hind paws and results were therefore combined to one data point.

Investigation of scaled stride length (Figure 2D) demonstrated a significant age effect [*F*_(2,174)_ = 5.51, *p* = 0.005]. At young and middle age, an early genotype effect was present [young: *p* = 0.0017, *F*_(2,60)_ = 7.110; middle age: *p* = 0.0425, *F*_(2,55)_ = 3.346] with significantly increased stride length in female, hom animals at young age (wt vs. hom *p* < 0.0001, het vs. hom *p* < 0.0001) and a clear trend toward the same effect in middle age, hom males (het vs. hom *p* = 0.0529). At young age, significant interaction between factors sex × genotype was observed [*p* = 0.0001, *F*_(2,60)_ = 10.42] with significant *post hoc* effect between female and male, hom animals (*p* = 0.0002). A significant longer stride length in female compared to male animals was detected beginning with middle age [middle age: *p* = 0.0127, *F*_(1,55)_ = 6.491; adult: *p* = 0.0003, *F*_(1,55)_ = 14.75] demonstrating a trend between middle aged wt animals (*p* = 0.0629) and significant difference within the wt and the hom group at adulthood (wt: *p* = 0.0459; hom: *p* = 0.0235).

Swing speed was also measured (Figure 2E) and showed similar alterations as observed in stride length. Significant decrease over age was measured [*F*_(2,181)_ = 11.41, *p* < 0.001]. At young and middle age, significant genotype effects were measured [young: *p* = 0.0006, *F*_(2,60)_ = 8.510; middle age: *p* = 0.0155, *F*_(2,60)_ = 4.508] with accelerated swing speed in female, hom animals at young age (wt vs. hom: *p* < 0.0001; het vs. hom: *p* < 0.0001). At young age, significant between-factor-interaction was computed [*p* = 0.0001, *F*_(2,60)_ = 10.73] with faster swing speed in hom, females compared to male counterparts (*p* = 0.0014). Differences between both sexes, were already present at middle age [*p* = 0.0672, *F*_(1,54)_ = 3.490] becoming statistically significant at adulthood [*p* = 0.0088, *F*_(1,56)_ = 7.366].

The Regularity Index (Figure 2F) reflects the number of normal step sequence patterns in relation to the total number of paw placements and approaches 100 % under physiological conditions, as reflected by young F344tgHD rats in this experimental setup. Overtime a slight, but significant decrease could be observed [*F*_(2,177)_ = 4.3109, *p* = 0.015]. At middle age, a sex dependent difference [*p* = 0.0384, *F*_(1,53)_ = 4.510] was measured with significant *post hoc* in the hom group (*p* = 0.0345). At adult age, rats displayed significant genotype effects [*p* = 0.0326, *F*_(2,56)_ = 3.641] with step regularity deficits in hom males (wt vs. hom *p* = 0.482, het vs. hom *p* = 0.0264).

The Coefficient of variance (CoV) reflects the intraindividual variability and is used to evaluate gait quality in patients. The CoV for stride length (Figure 2G) showed significant decrease over time [*F*_(2,185)_ = 5.6907, *p* = 0004], while CoV for body speed/velocity (Figure 2H) demonstrated a clear trend toward the same effect [*F*_(2,187)_ = 2.5867, *p* = 0.078]. Neither genotype nor sex exerted an impact on the CoV for stride length or body speed in the F344tgHD rat model.

### 3.3 Acoustic startle response

#### 3.3.1 Male F344tgHD show elevated startle reaction compared to females with a transiently reduced reaction in hom animals at middle age

**FIGURE 3:**
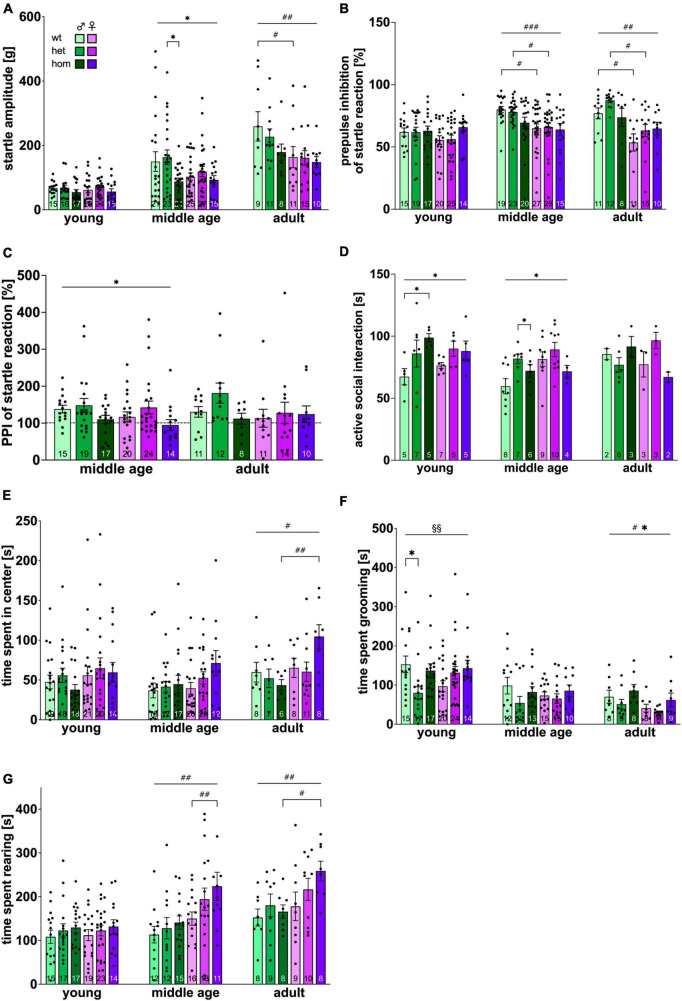
Emotional status of F344tgHD rats. Emotion-driven parameters obtained with the acoustic startle system **(A–C)**, the social interaction test **(D)** and the open field test **(E–G)** were investigated. **(C)** PPI of acoustic startle reaction at middle and adult age is presented as percentage of reaction shown at young age (dotted line). Data is presented as mean ± SEM. Significant statistical effects are labeled as follows: * genotype, ^#^ sex, ^§^ interaction. The investigated n is indicated at the bottom of each bar. The legend is depicted in **(A)**. The ASR amplitude is provided in gram (g).

The ASR test was conducted to measure sensorimotor reflexes of the animal toward acoustic stimuli. The ASR approach is of high face validity and is measured via whole-body ballistic movement in rodents and electromyographic recordings in humans (Gómez-Nieto et al., 2020). In all animals, the intensity of startle responses showed a positive correlation with corresponding impulse intensity at all ages. Full data set can be found in the supplements (Supplementary Figure 4A). Figure 3A depicts the animals’ reaction to 115 dB pulses. A significant increase in the animals’ startle reaction was evident over time [*F*_(2,300)_ = 46.082, *p* < 0.001]. No difference could be observed between sex, nor genotype at young age. At middle age, a trend toward increased startle response in male animals compared to females [*F*_(1,126)_ = 3.3348, *p* = 0.0696] was present and a main genotype effect was measured [*F*_(2,126)_ = 3.380, *p* = 0.0372] with significantly reduced startle reaction in hom compared to het males (*p* = 0.016) and almost significantly to wt littermates (*p* = 0.0676). At adult age, significantly higher startle amplitudes for males [*F*_(1,58)_ = 7.260, *p* = 0.0092] were revealed with no significant *post hoc* comparison. Interestingly, no genotype dependent difference could be detected in females at any investigated age point.

#### 3.3.2 Male F344tg show more pronounced inhibition of ASR by a pre-pulse stimulus than females

The inhibition of the acoustic startle response via pre-pulse stimulation was analyzed in a subsequent approach. Again, the intensity of the pre-pulse stimulus correlated with the magnitude of inhibition of the startle response at all ages tested (Supplementary Figure 4B). The inhibition of the startle response for an 80 dB pre-pulse is depicted in Figure 3B. A significant increase in PPI of ASR was measured over time mainly caused by the male group [*F*_(2,305)_ = 10.377, *p* < 0.001]. An increased startle inhibition in males was revealed beginning with middle age [*F*_(1,126)_ = 12.95, *p* = 0.005] and persevering until adulthood [*F*_(1,62)_ = 8.286, *p* < 0.0055]. Male wt and het rats startled significantly less than female animals of the respective genotype (middle age: wt *p* = 0.013, het *p* = 0.0294; adult: wt *p* = 0.0356, het *p* = 0.0135). No significant genotype could be measured at any age point. As additional approach, PPI was calculated as fold change from the initial baseline (young age) (Figure 3C). While wt and het animals startled less after a pre-pulse at middle age in proportion to their startle reaction at young age, hom animals remained on the same level, resulting in significant genotype differences at middle age [*F*_(2,103)_ = 4.519, *p* = 0.0132] with no significant *post hoc* comparison. No significant age effect was detected.

### 3.4 Male homozygous F344tgHD rats display early anxiety-reduced behavior in the social interaction test

Assessment of anxiety levels were conducted with the social interaction test. The average time per active interaction between each rat pair was evaluated (Figure 3D). A clear trend toward an age effect was measured [*F*_(2,89)_ = 2.763, *p* = 0.069]. No significant difference between sexes could be measured at any investigated age point, although a trend for increased female interaction was present at middle age [*F*_(1,40)_ = 3.518, *p* = 0.068]. A significant genotype effect was detected at young and middle age [young: *F*_(2,28)_ = 4,720, *p* = 0.0171; middle age: *F*_(2,40)_ = 4,04, *p* = 0.0252] with an indicative gene-dosage dependency at young age. *Post hoc* comparison revealed increased socio-positive interaction in tg animals (young: wt vs. hom *p* = 0.0193; middle age: het vs. hom *p* = 0.0316).

### 3.5 Open field assay demonstrates sex dimorphism at older age points in emotion-driven parameters

The open field test takes advantage of the animal’s subjective conflict between the drive to explore and aversion to the exposure toward a brightly lit open arena, hence the assay provides information on spontaneous locomotion and anxiety-related parameters. All animals spent the majority of the test period close to the peripheral walls of the arena (thigmotaxis) and displayed sufficient exploratory behavior (Supplementary Figure 5A).

The time spent in the center of the arena (Figure 3E) showed a trend for an increase over time [*F*_(2,259)_ = 2.609, *p* = 0.076]. At young and middle age, animals spent equal time in the center of the arena regardless of their sex or genotype. At adulthood, females stayed more often in the arena’s center than males [*F*_(1,42)_ = 5.767, *p* = 0.0208] with hom females spending the most time in the center and significantly differing from hom males (*p* = 0.0078). Time spent in the center of the arena was additionally calculated as percentage of the total time spent in locomotion (Supplementary Figure 5B), which supports the findings described above, though not reaching statistical significancy.

Analysis of grooming behavior (Figure 3F) confirmed a significant age effect with a decrease over time [*F*_(2,236)_ = 25.618, *p* < 0.001]. Significant interaction between factors “sex” and “genotype” was present at young age [*F*_(2, 101)_ = 5.063, *p* = 0.008] with significant less grooming in het compared to wt males (*p* = 0.0164). Animals of adult age displayed significant sex- and genotype-dependent overall effects [sex: *F*_(1,47)_ = 5.470, *p* = 0.0237; genotype: *F*_(2,47)_ = 3.439, *p* = 0.0404] with males grooming more than females and hom animals more than het and wt littermates.

Rearing showed significant increase over time [*F*_(2,238)_ = 18.512, *p* < 0.0001] (Figure 3G). Beginning with middle age, females spent significantly more time rearing than males [middle age: *p* = 0.0013, *F*_(1,78)_ = 11.21; adult: *p* = 0.0145; *F*_(1,46)_ = 6.458] with significant *post hoc* between homozygous animals (middle age: *p* = 0.0429; adult: *p* = 0.0420).

Hom females showed ophistotonus-like head movements at the oldest time point investigated, as described for adult SPRDtgHD rats (Cao et al., 2006). These choreiform movements were detected in 6 out of 10 hom females with 5.5 ± 0.5627 events per 15 min observation time (Supplementary Figure 6). No such anomalies were seen in het females, nor male F344tgHD animals.

### 3.6 Automated phenotyping in a home cage-like environment reveals sex dimorphism in general activity and metabolic parameters and increased locomotion in adult homozygous females

Detection of ethological behaviors, their circadian patterns, as well as corresponding energy expenditure (derived from indirect calorimetry) were performed in a home cage-like environment within the PhenoMaster System (TSE Systems GmbH, Germany). All animals’ behaviors displayed typical circadian rhythmicity (data not shown).

**FIGURE 4:**
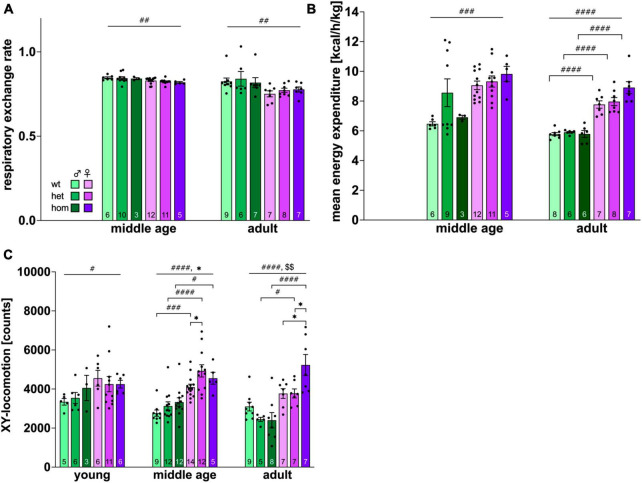
Activity and metabolic parameters obtained via automated phenotyping of F344tgHD rats. **(A)** Respiratory exchange rate, **(B)**: energy expenditure, **(C)**: locomotion in *X* and *Y*-axis. Data is presented as mean ± SEM. Hashtags indicate significant statistical effects between sexes (^#^*p* < 0.05, ^##^*p* < 0.02, ^###^*p* < 0.001, ^####^*p* < 0.0001). Asterisks indicate significant genotype differences (*p < 0.05). Paragraphs indicate significant interaction between factors “sex” and “genotype” (^§§^ < 0.02). The investigated n is indicated at the bottom of each bar. The legend is depicted in **(A)**.

Energy expenditure (EE) and respiratory exchange rate (RER) were measured only at middle and adult age due to technical issues. Both parameters showed an age-dependent decrease [EE: *F*_(2,85)_ = 23.934, *p* < 0.001, RER: *F*_(2,88)_ = 12.570, *p* < 0.001]. The RER gives information about the main energy source with values around 0.8 for a fat-based and >0.8 for carbohydrate-based metabolism. All groups displayed fat-based calorimetry with values around 0.8. RER was significantly lower within the female group at both age points [middle age: *F*_(1,41)_ = 9.489, *p* = 0.0037; adult: *F*_(1,38)_ = 9.770, *p* = 0.0034] without significant *post hoc* comparison (Figure 4A).

In contrast, male animals demonstrated significantly lower EE than females at middle age and adulthood [middle age: *F*_(1,42)_ = 8.405, *p* = 0.0059; adult: *F*_(1,36)_ = 136.8, *p* < 0.0001] with no significant *post hoc* at middle age but within all genotypes at adult age (wt: *p* < 0.0001, het: *p* < 0.0001, hom: *p* < 0.0001) (Figure 4B). No genotype effect could be shown for EE or RER.

Investigation of locomotion (Figure 4C) in x- and y-axis revealed a significant age effect [*F*_(2,141)_ = 3.198, *p* = 0.044] and higher exploratory activity in females throughout testing [young: *F*_(1,31)_ = 4.383, *p* < 0.0446; middle age: *F*_(1,58)_ = 52,18, *p* < 0.0001; adult: *F*_(1,37)_ = 33.96, *p* < 0.0001]. Significant *post hoc* comparison was present between all genotypes at middle age (wt *p* = 0.0004; het *p* < 0.0001; hom *p* = 0.0110) and between transgenic animals at adult age (het *p* = 0.0446; hom *p* < 0.0001). Genotype differences were additionally observable at middle age [*p* < 0.0253, *F*_(2,58)_ = 3.919] with increased activity in het females compared to controls (*p* = 0.0196). An interaction between factors genotype and sex could be shown in the adult group [*F*_(2,37)_ = 5.855, *p* = 0.0062] with significant higher interaction of hom females compared to wt (*p* = 0.0107) and het (*p* = 0.00111) animals. Additionally, a trend for increased locomotion of transgenic animals was detected at adult age [*F*_(2,37)_ = 2.918, *p* = 0.0666].

### 3.7 Polyglutamine-containing aggregates visible at 8 months of age

**FIGURE 5:**
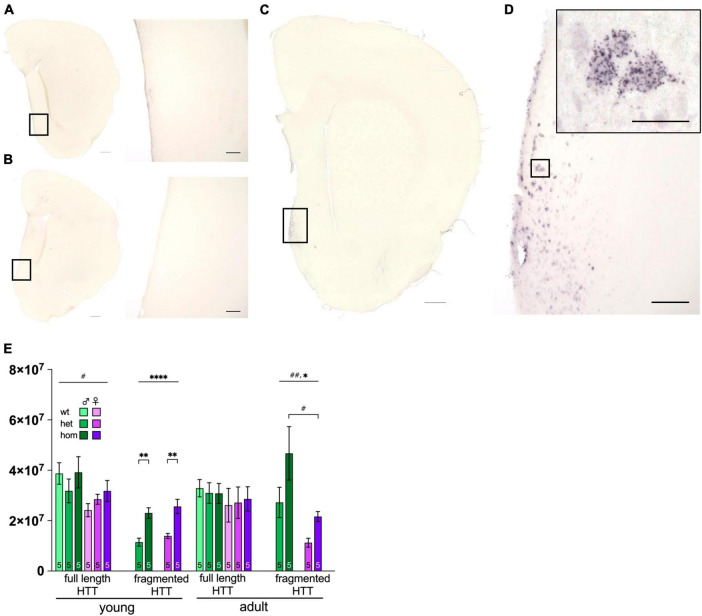
Huntingtin protein distribution and levels in F344tgHD rats. Immunohistological detection of mHTT via anti-polyQ antibody 1c2 in 8 months old F344tgHD male is exemplarily depicted for one hemisphere of wt **(A)**, het **(B)** and one hom **(C)** animal including a magnified portion of the highlighted area **(D)**. Scales in **(A–C)** =̂ 500 μm, scale in **(D)** =̂ 100 μm and in magnified area =̂ 25 μm. **(E)** Protein level of full length and transgenic (fragmented) HTT were quantified with anti-HTT antibody Mab2166 in striatum enriched samples at young and adult age in F344tgHD males and females. Data is presented as mean ± SEM. Hashtags indicate significant statistical effects between sexes (^#^*p* < 0.05, ^##^*p* < 0.02, ^###^*p* < 0.001, ^####^*p* < 0.0001). Asterisks indicate significant genotype differences (*p < 0.05). Paragraphs indicate significant interaction between factors “sex” and “genotype” (^§§^ < 0.02). The investigated n is indicated at the bottom of each bar.

Neurohistological examination of F344tgHD revealed first polyglutamine-containing aggregates at the age of 8 months (Figures 5C, D). Aggregates were detectable in medial forebrain nuclei of hom animals. No difference in the appearance of HTT aggregates was observed between sexes. Brains derived from wt and het animals did not show 1C2-antibody-specific immunoreactivity in the investigated coronal section (Figures 5A, B).

### 3.8 Protein levels of mHTT reflect the animals’ genotype

Western blot analyses of total HTT were performed on striatum enriched tissue to ensure a gene-dosage effect in protein levels (heterozygous vs. homozygous) while excluding sexual dimorphism (Figure 5E and Supplementary Figure 7). Full length HTT was increased in young females compared to males [*F*_(1,24)_ = 5.972, *p* = 0.0223], while both sexes showed equal expression at adult age. As anticipated, transgenic (fragmented) HTT was expressed with a significant gene-dose effect at both ages [young: *F*_(1,16)_ = 33.90, *p* < 0.0001; adult: *F*_(1,16)_ = 5.718, *p* = 0.0294] with significant *post hoc* between het and hom animals in both sexes at young age (young het vs. hom: male *p* = 0.0017; female *p* = 0.0015). Additionally, females expressed less transgene at adult age compared to males [*F*_(1,16)_ = 0.0046, *p* = 0.0046] with significant *post hoc* comparison in hom animals (*p* = 0.0118).

### 3.9 No striatal volume decrease but reduction of DARPP32-positive cells in tg F344tgHD animals at 8 months

**FIGURE 6:**
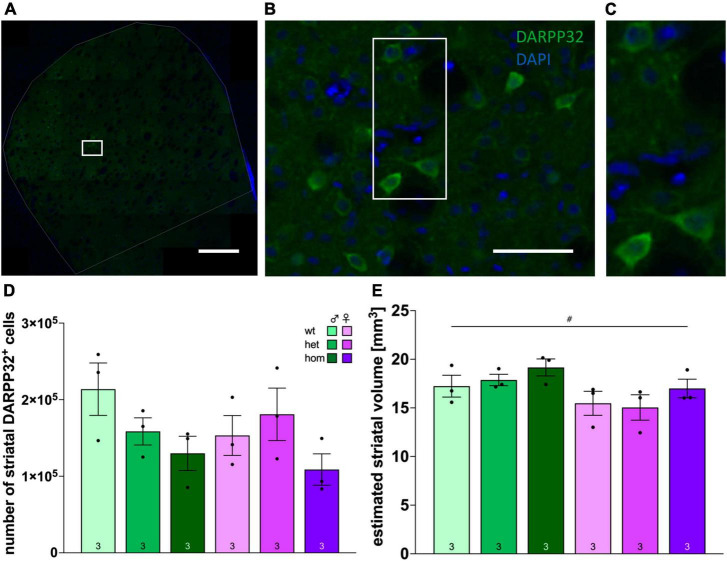
Stereological investigation of the number of DARPP32-positive cells and striatal volume in F344tgHD rats. Representative image of histological detection of DARPP32-positive cells in the striatum **(A)** and magnifications **(B)**, **(C)** as used for stereological analysis. **(D)** Numbers of DARPP32-positive cells in the striatum of tg female and male F344tgHD rats and control animals at adult age determined by stereological analysis with the optical fractionator method. **(E)** Volume estimation of the striatum of tg female and male F344tgHD rats and control animals at adult age determined by stereological analysis according to Cavalieri’s principle. Data is presented as mean ± SEM. Significant statistical effects between sexes are labeled with ^#^. The investigated n is indicated at the bottom of each bar. The legend is depicted in **(C)**.

Stereological quantification of the number of DARPP32-positive cells in the striatum was performed in a subset of 8 months old F344tgHD rats (*n* = 3/group) (representative staining in Figures 6A–C) and revealed no significant difference in the absolute number of neurons (Figure 6D). Yet, looking at the relative numbers, a reduction of around 26 % in heterozygous and nearly 40% in homozygous males and 30% in homozygous females was evident compared to the respective wt controls.

At the same time, no pathological change in striatal volume was detected yet (Figure 6E), and males exhibited significantly larger portions of striatum than female rats [*F*_(1,12)_ = 7.915, *p* = 0.0156].

## 4 Discussion

This is the first comparison of behavioral and neuropathological changes in both sexes of the F344tgHD rat model of HD and the first study designed to directly evaluate a prodromal-like phase in this model. We found first subtle behavioral changes in F344tgHD rats already at young age and significant differences between sexes especially in the motor and sensorimotor domain associated with decreased numbers of DARP32^±^cells in tg animals compared to wt controls at 8 months of age.

The F344tgHD model is one of only three available rat models for HD (SDtgHDCAG_*n*51*trunc*_; F344tgHDCAG_*n*51*trunc*_; BACHD_*CAGCAAn*98_) and constitutes a congenic version of the previous SPRDtgHD model. Higher reproducibility and reliability are ensured by inbreeding of the F344tgHD rats (Festing, 2010) and their smaller body size is advantageous for imaging techniques (Urbach et al., 2014; Plank et al., 2018). The F344 background further qualifies this model especially for research on stress-related neuroendocrinology and -inflammation (Vodička et al., 2020).

Male representatives have been previously characterized at symptomatic age by our group demonstrating the major key aspects of the adult-onset form of HD (von Hörsten et al., 2003; Plank et al., 2018). A thorough characterization of the rat equivalent to the human prodromal phase, currently in focus for early interventional treatments, as well as investigation of zygosity effects and potential sex dimorphism, was missing so far.

Hence, we performed a comprehensive, longitudinal, cross sectional, in-depth analysis of female and male 1–8 months old F344tgHD rats. We thereby aim at increasing the validity and translatability of future studies while simultaneously causing a decrease in the number of used animals in the sense of the 3R principle “refine.”

### 4.1 General health and reproduction

The F344tgHD rat line is of general health and does not develop any impairments that could affect its behavioral characterization at early age. In line with published data on older, male F344tgHD rats and the previous SPRDtgHD model (Kirch et al., 2013; Urbach et al., 2014; Plank et al., 2018) neither female nor male transgenic animals showed significant differences in weight compared to control animals. As expected, females had physiologically lower body weights beginning already at 4 weeks of age. Intra-home cage ethophysiological analysis using an automated PhenoMaster system revealed no difference in food ingestion when corrected for body size. However, the increased energy expenditure seen in females might contribute to the observed weight difference. A significant connection between these parameters has been reported in different mouse strains (König et al., 2020).

Analysis of the genotype ratio among F344tgHD descendants revealed slight deviation from the Mendelian distribution known for a monogenetic, autosomal dominant disease, like HD. The discrepancy manifested especially in female animals with decreased numbers of hom descendants. Although these deviations are not in a magnitude calling for major impairment of embryonic development in the F344tgHD rat model, our data might indicate pre-natal decease of preferentially hom animals due to yet unknown factors and we consider them as important information for future biostatistical planning of studies.

### 4.2 Investigations of molecular HD hallmarks

Investigation of HD neuropathological hallmarks was performed in the striatum of F344tgHD rats to examine their occurrence in relation to behavioral abnormalities over time. Aggregates containing mHTT were detectable at 8 but not 5 months of age in hom F344tgHD rats. In the SPRDtgHD model, first aggregates were already seen at 6 months (Nguyen et al., 2006), indicating a slower disease progression in the congenic F344tgHD model.

Male F344tgHD rats showed pronounced striatal cell loss with a gene-dosage effect, i.e., smaller cell numbers in hom compared to het animals. While hom females also showed a decrease in the number of DARPP32^±^cells compared to wt controls, het females demonstrated an unexpected increase (+10 %). Besides the underlying zygosity effect, compensatory mechanisms, e.g., via 17ß-estradiol (Bode et al., 2008) might be causal for this deviation.

In general, females showed less distinct striatal cell degeneration compared to the respective male group. This sex-effect might be attributed to the physiological higher levels of 17ß-estradiol and their neuroprotective effect, studied intensely in neurodegenerative diseases (reviewed: Dhandapani and Brann, 2002). In the SPRDtgHD rat model, Bode et al. (2008) showed a direct correlation between 17ß-estradiol level and number of DARPP32^±^cells supporting the described findings in F344tgHD rats.

The possible reasons for the measurable cell death in het males without the detection of aggregates in the rostral part of the striatum, and the relatively low number of aggregates in hom animals, comprise a variety of hypothesis: First, aggregates were shown to be unequally distributed throughout the brain, with more aggregates in the neocortex compared to the striatum and a gradient within the later toward more aggregates in caudal direction in human patients (Kuemmerle et al., 1999). Second, current literature suggests that it is rather the structure of mHTT aggregates that predicts cell death than their abundance. A study in R6/2 mice, e.g., showed that loop-structured aggregates in the striatum have highly neurotoxic properties, while ß-sheet containing aggregates in the cerebellum are only mildly toxic (Nekooki-Machida et al., 2009). Third, various studies report a neuroprotective effect by the formation of aggregates, sequestering mHTT in the cell (reviewed: Bäuerlein et al., 2020).

### 4.3 Emotionality

Socio-positive behavior in pairs of rats with the same genotype and unfamiliar to each other, were measured with the SI test as the time spent in active social interaction in a novel environment (File and Seth, 2003). Transgenic males demonstrated an early anxiety-reduced phenotype in the form of increased social interaction at young and middle age, confirming previous results (Plank et al., 2018) and matching reports in the SPRDtgHD model (von Hörsten et al., 2003; Nguyen et al., 2006; Bode et al., 2008; Kirch et al., 2013).

Female F344tgHD did not show such clear anxiolytic-like phenotype in the SI test. In the OF test, though, females showed more rearing beginning with middle age, a behavior comprising both an emotional (exploration) and a motoric component (ability to balance on hind paws). Hom females reared more than het littermates, indicative of a gene-dosage effect. They also spent more time in the center of the arena than other animals in this study at adult age, adding to the picture of a less anxious phenotype. It appears that females experience emotional disturbances at older age points and that this domain is affected later than in males.

The impact of intersessional habituation on the corresponding test construct validity is of particular relevance for the interpretation of data derived from the OF test, as “exposure to novelty” as the underlying behavioral construct is gradually lost upon repeated exposure. Thus, loss of novelty of the testing procedure and environment is unavoidable in such experimental designs, if the study is designed longitudinally. In this study, the same animals were repeatedly tested from 1 to 8 months of age, which includes certain habituation effects over time. However, the data presented here do not directly indicate major habituation effects on the OF performance, e.g., no observable decrease in exploratory activity (Supplementary Figure 5A).

### 4.4 Sensorimotor function

A mild impairment in sensorimotor function in form of a decreased ASR was detected in hom F344tgHD males beginning with middle age. Since no hearing deficits were observed and response to acoustic stimuli was normal at young age, basal deficit in sensorimotor gating can be ruled out. Our results correspond with data on other mouse and rat HD models, e.g., BacHD rats (Abada et al., 2013) and R6/2 mice (Carter et al., 1999) who also measured decreased startle reaction in transgenic animals. The additionally observed sexual dimorphism toward higher startle amplitudes in male animals matches data published on other strains, e.g., Wistar (Lehmann, 1999), Long Evans and SPRD rats (Faraday, 2002).

In contrast to published literature, no longitudinal habituation in form of a decreasing startle response over the inspected time course could be observed, stating no influence of the F344tgHD background nor a possible age related hyporeactivity (Gómez-Nieto et al., 2020).

Presence of an acoustic pre-stimulus (PPI) showed transient genotype-associated effects in the ASR feedback-regulation at middle age when normalized to the initial baseline PPI. While wt and het animals showed up to 50% higher PPI at middle age compared to their reaction at young age, hom animals of both sexes remained on the same level. This matches the early anxiolytic-like phenotype of hom F344tgHD animals observed in the SI and OF test. The PPI is exerted by a sensorimotor circuitry via the reticular formation and with participation of several different neurotransmitters. Deficits are often observed in symptomatic HD patients (Swerdlow et al., 1995), while the previous SPRDtgHD rat model showed absence of these (Höhn et al., 2011; Urbach et al., 2014).

The clear sexual dimorphism found in F344tgHD rats regarding the PPI is in line with findings in healthy human subjects, where women show less PPI than men (Swerdlow et al., 1993; Kumari et al., 2003). However, not only sex in general affects PPI measures in humans, but intraindividual changes dependent on the female estrous cycle were detected as well (Swerdlow et al., 1997). To our knowledge, this has not been intensely studied in rodents so far, but our results do not indicate such effect, since no alterations of the PPI were observed between pre-pubescent and pubescent females nor does the magnitude of inter-individual variation suggest such phenomenon.

Although ASR and PPI function via involuntary sensorimotor gating processes, the individual’s emotional status is a known side factor influencing this reflex (Koch and Schnitzler, 1997; Grillon and Baas, 2003). In patients with stress-induced psychological diseases the startle reaction is a commonly examined parameter (Swerdlow, 1994; Kaviani et al., 2004; Ray et al., 2009) and the ASR amplitude can be increased by various stressors in the SPRD rat strain (Acri, 1994; Garrick et al., 1997; Servatius et al., 2000; Faraday, 2002). Hence, the anxiety-reduced phenotype in F344tgHD males observed in the SI approach is further supported by the decrease in ASR amplitude in this study.

### 4.5 Motor function

Motor coordination, balance, and gait were analyzed using the classical RotaRod test as well as the CatWalk system. Complementary data were obtained with the intra-home cage-like Phenomaster analysis.

On the RotaRod, significantly better performance of all females was detected already at young age. This observation has also been described for Sprague Dawley rats (Bagg et al., 2017) and might be, at least to some extent, explainable by the existing sex dimorphism in body weight. Boakes et al. (1999) showed that activity on a running wheel can be increased in male rats through weight loss. In consistency with the RotaRod data, females were also more active in the OF paradigm as well as in the Phenomaster home cage system, where potential influence through the experimental setup (presence of experimenter, preceded handling, etc.) can be ruled out. The observed activity profile of female individuals also matches the comparatively higher energy expenditure measured in the automated Phenomaster system. The later seems to be physiologically increased in female rodents compared to male littermates (König et al., 2020).

No obvious gene-dosage effects could be detected in RotaRod performance of neither sex, but tg males demonstrated increased coordination skills at young age corresponding with previous data from 2 months old male F344tgHD (Plank et al., 2018) and 1 month old SPRDtgHD rats (Nguyen et al., 2006). “Hypercompensation” through increased neuronal excitability has been discussed as causal, as seen in other motor diseases like ALS, but still needs to be further characterized (Nguyen et al., 2006). The expanded CAG repeat stretch in the *Htt* gene supposedly constitutes an evolutionary benefit in form of increased fitness to the carrier (Eskenazi et al., 2007; Mühlau et al., 2012; Schultz et al., 2021) which might additionally take effect here.

In addition to the classical RotaRod approach, we addressed the demand made in Plank et al. (2018) to apply an additional, more sensitive method to investigate motor function and gait. The CatWalk assay can measure over 250 parameters and allows a more holistic investigation of locomotion compared to the classical RotaRod approach (Vandeputte et al., 2010; Timotius et al., 2023). Clear differences were detected between both sexes of the F344tgHD model, as already observed with the RotaRod approach.

Increased values for stride length and swing speed measured in females are in line with a general higher activity as described above. Male animals tended to display a higher BOS throughout the experimental phase, an observation also made in Sprague Dawley rats (Bagg et al., 2017) and most probably accountable their higher body weight compared to females.

In regard to genotype-related differences, subtle changes could be detected already at the earliest investigated time point. Hom females displayed increased velocity, swing speed and stride length with larger BOS in hind paws. They also moved significantly more in the PM paradigm and showed opisthotonos head movements in the OF arena. The later has also been described for older SPRDtgHD females (Cao et al., 2006) and is a unique observation in HD rodent models, reminiscent of the choreatic movements in human HD.

Our observations on a hyperkinetic phenotype in female F344tgHD beginning with young age matches data of other HD rat (Nguyen et al., 2006; Vandeputte et al., 2010) and mouse models (Menalled and Chesselet, 2002). In patients, hyperkinetic movements are observable in the early phase of the disease (Delval et al., 2006) with increased stride length in one out of six HD patients (Reynolds et al., 1999).

A higher BOS has also been described for symptomatic HD patients (Rao et al., 2008; Pyo et al., 2017) and might be a compensatory mechanism for the increased instability through choreiform movements (Koller and Trimble, 1985).

Male tg F344tgHD showed less impairment in the motor domain as seen in the Catwalk analysis, besides an increased alteration in the step pattern (regularity index) at 8 months, also matching reports on HD patients (Pyo et al., 2017; Gaßner et al., 2020; Radovanović et al., 2020).

## 5 Conclusion

Taking into account the neuropathological status of the animals, where they present mHTT-containing aggregates and pronounced striatal cell loss with a gene-dosage effect at 8 months, male F344tgHD convincingly replicate a prodromal-like phase as seen in human HD patients with early emotional disturbances prior to an apparent motor phenotype and observable neuropathology.

In females the correlation between behavioral phenotype and neuropathology seems to be more complex. Hom females showed an early hyperactive motor phenotype with choreiform head movements, while subtle emotional disturbances became overt later. In contrast, het F344tgHD females did neither show a motor phenotype, nor significant cell loss, but instead an increase in the number of DARPP32^±^cells. Hence, het F344tgHD females present an unexpected delay of onset of HD symptoms.

In summary, our study shows that HD progression follows a sex-dependent course in the F344tgHD rat model with a clear prodromal-like phase in hom males and a relatively stronger motor phenotype in hom females. This characterization increases the validity and translatability of the model, contributing to the 3R principle “refine.” The described differences between female and male rats emphasize the importance of including sex as biological variable in preclinical studies to avoid a gender data gap and increase the translatability of animal models and the associated outcome of therapeutical studies thereon.

## Data availability statement

The original contributions presented in the study are included in the article/Supplementary material, further inquiries can be directed to the corresponding author.

## Ethics statement

The animal studies were approved by Government of Lower Franconia, Bavaria, Germany. The studies were conducted in accordance with the local legislation and institutional requirements. Written informed consent was obtained from the owners for the participation of their animals in this study.

## Author contributions

VR-W: Writing – original draft, Conceptualization, Data curation, Formal Analysis, Investigation, Methodology, Project administration, Visualization. JoH: Writing – review and editing. SM: Writing – review and editing. JuH: Data curation, Writing – review and editing. CS: Writing – review and editing. SH: Supervision, Writing – review and editing, Conceptualization, Data curation, Funding acquisition, Project administration, Resources.
