# Supplementary figures and images for: Gene-dosage- and sex-dependent differences in the prodromal-Like phase of the F344tgHD rat model for Huntington disease
Source: Front Neurosci. 2024 Feb 7;18:1354977. doi: 10.3389/fnins.2024.1354977 (PMC10879377; doi:10.3389/fnins.2024.1354977)

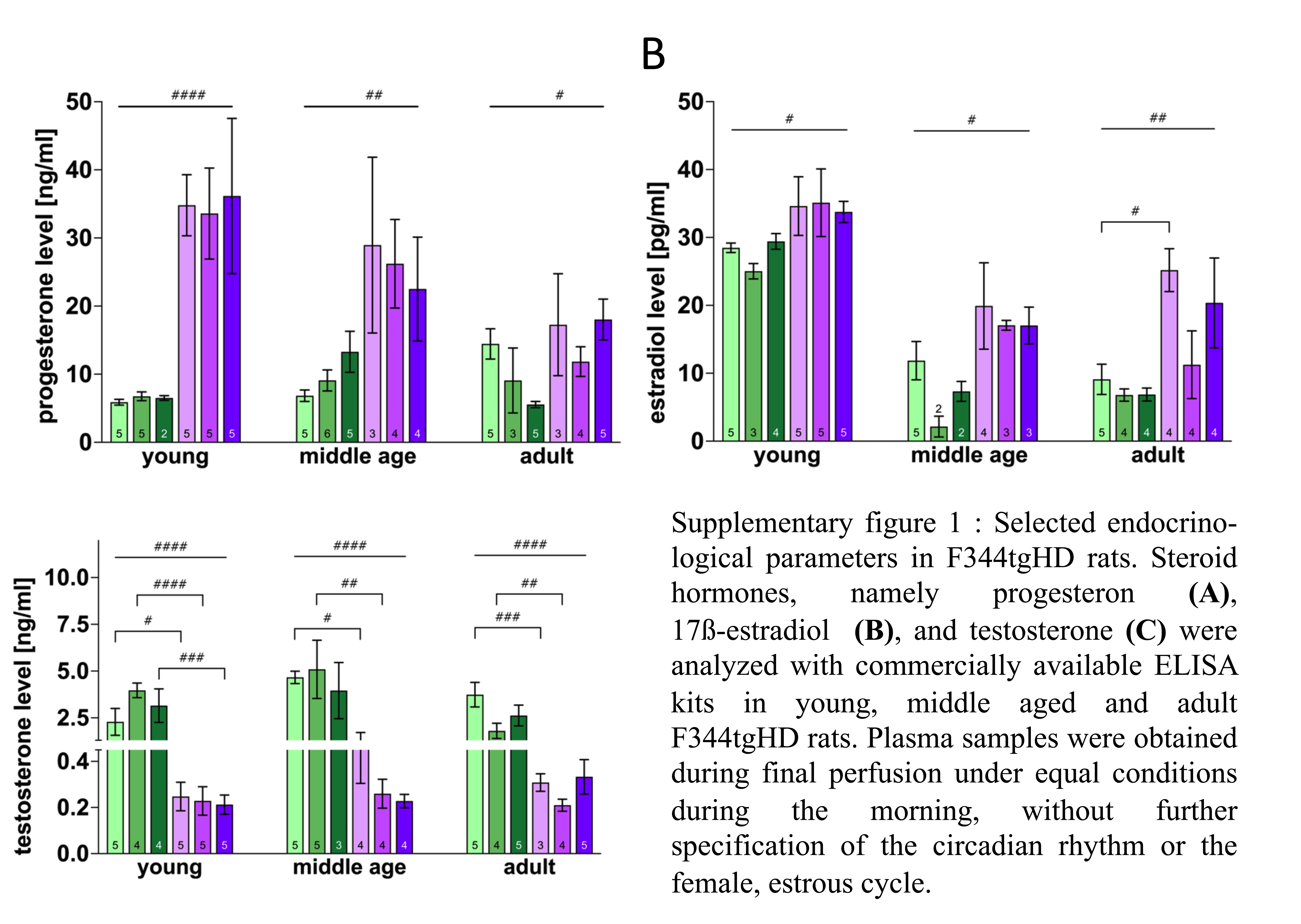

Supplement: Supplementary file 1 [file Image_1.tiff]

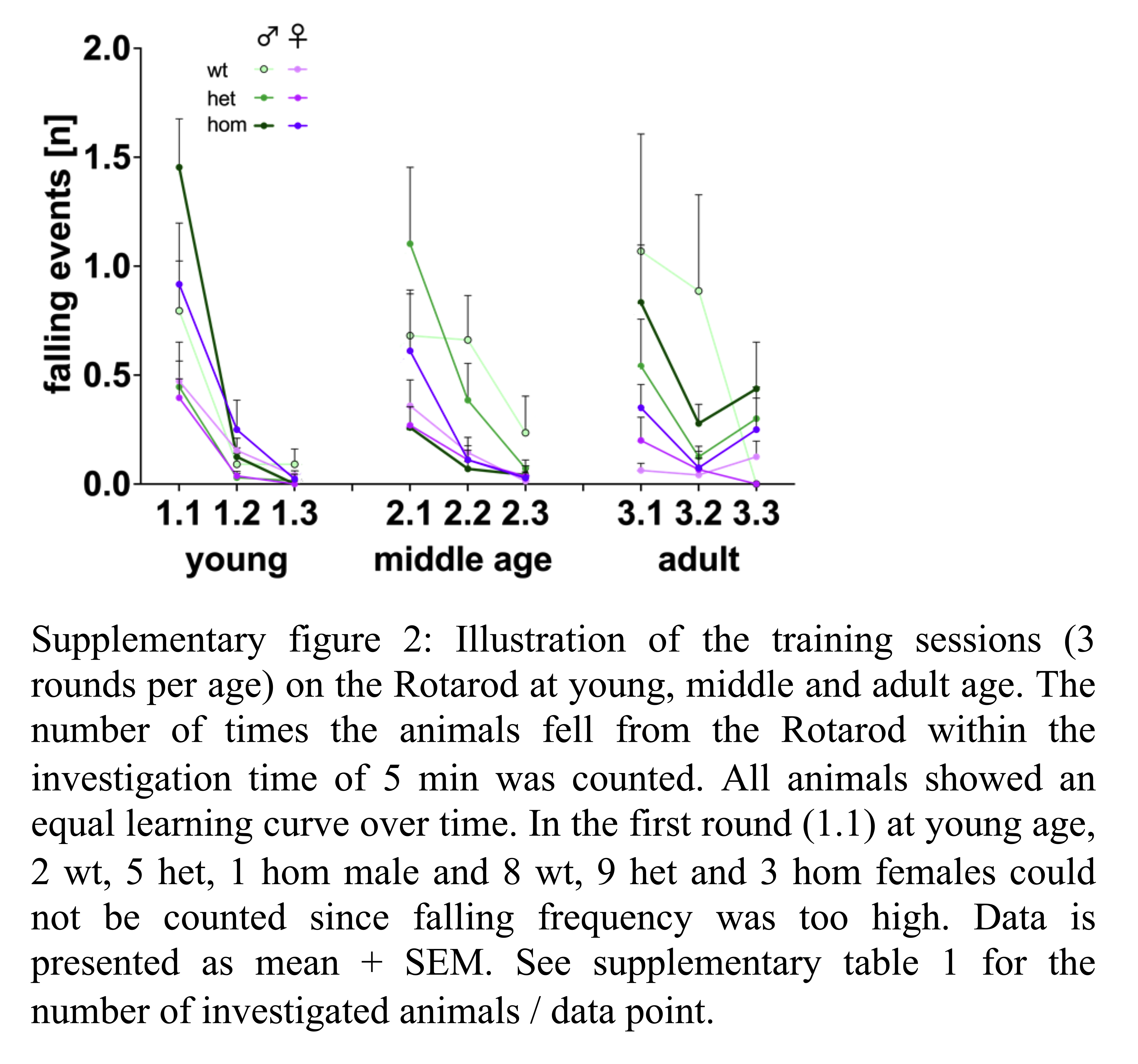

Supplement: Supplementary file 2 [file Image_2.TIFF]

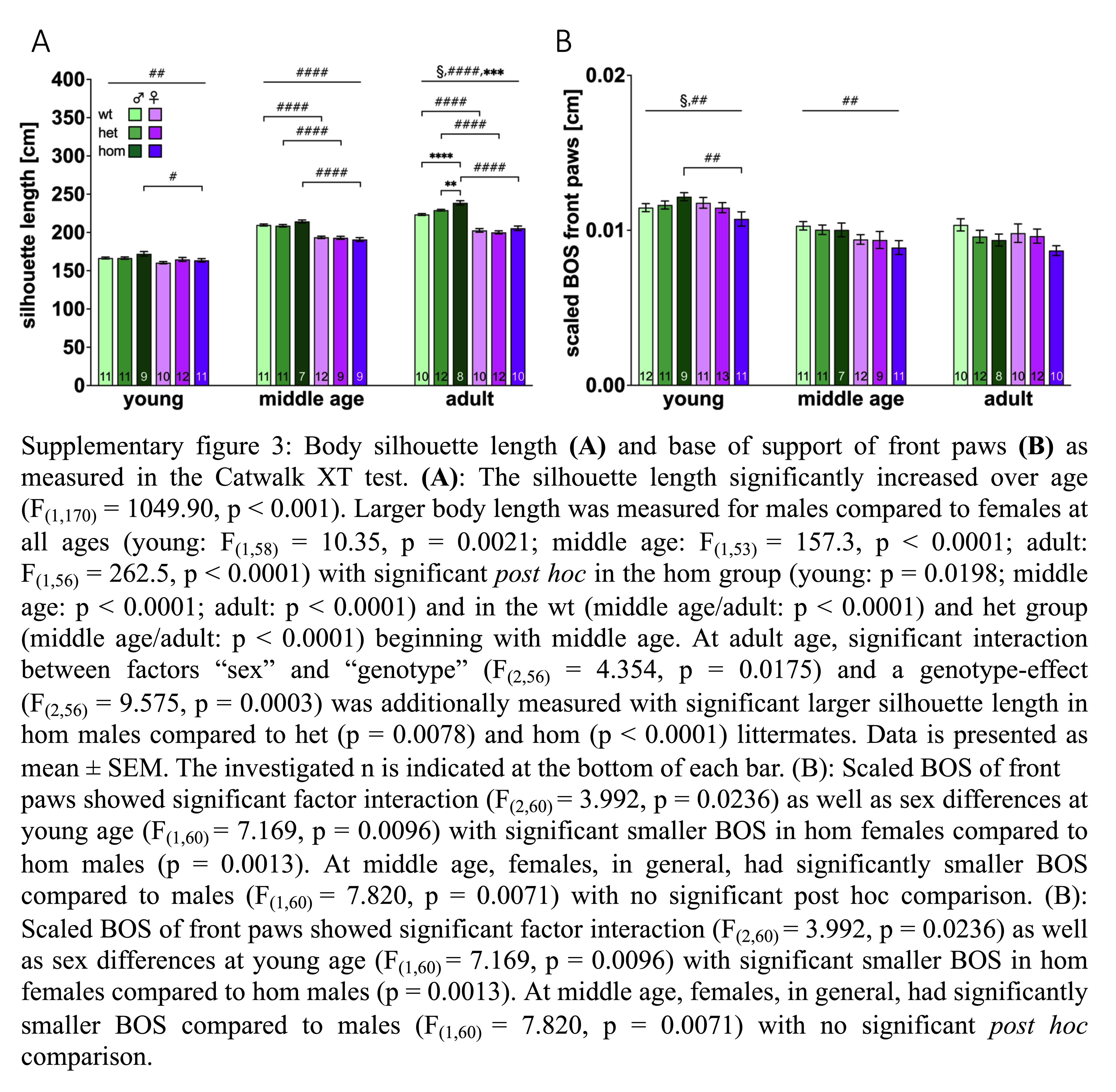

Supplement: Supplementary file 3 [file Image_3.tiff]

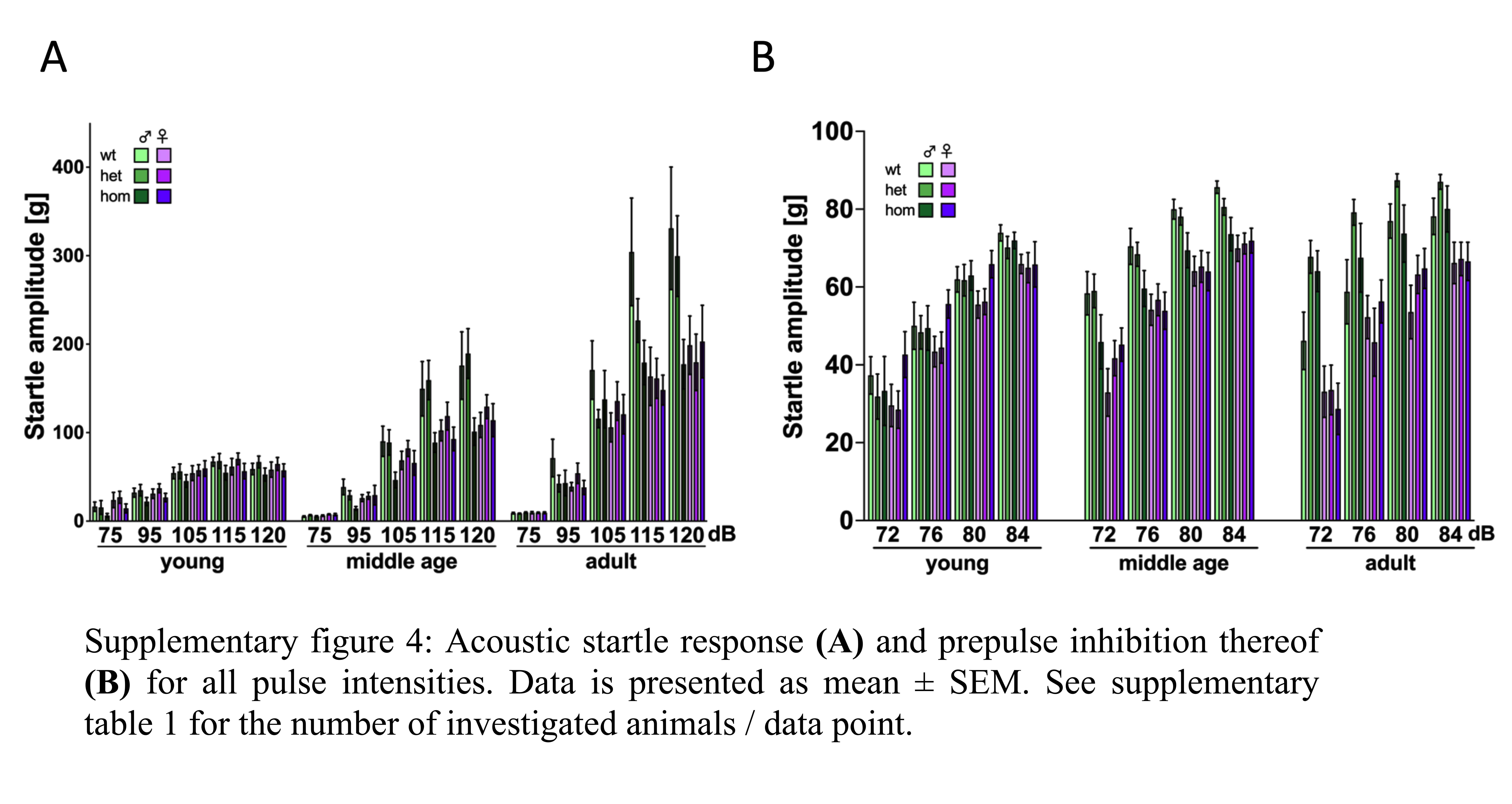

Supplement: Supplementary file 4 [file Image_4.TIFF]

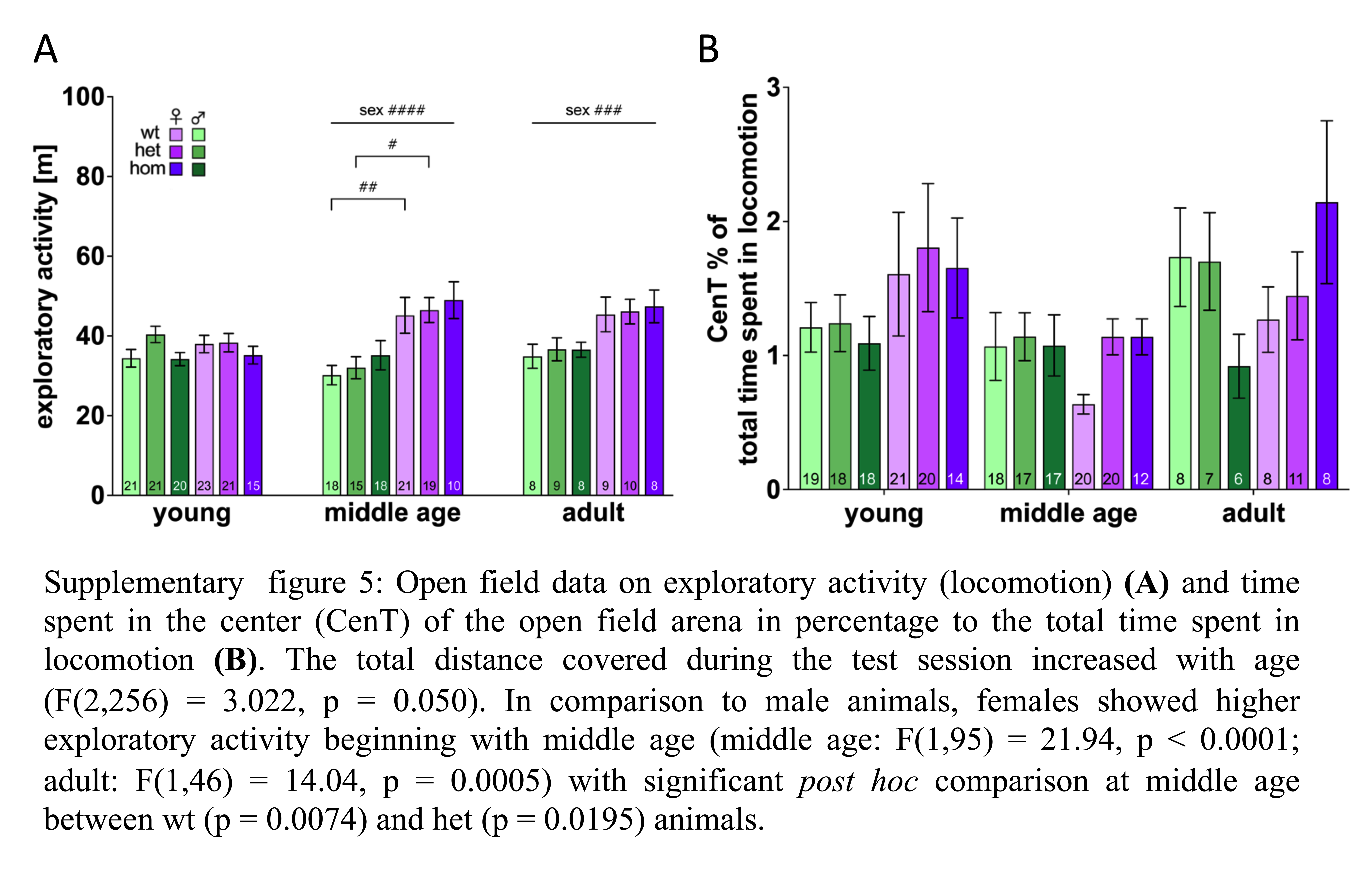

Supplement: Supplementary file 5 [file Image_5.TIFF]

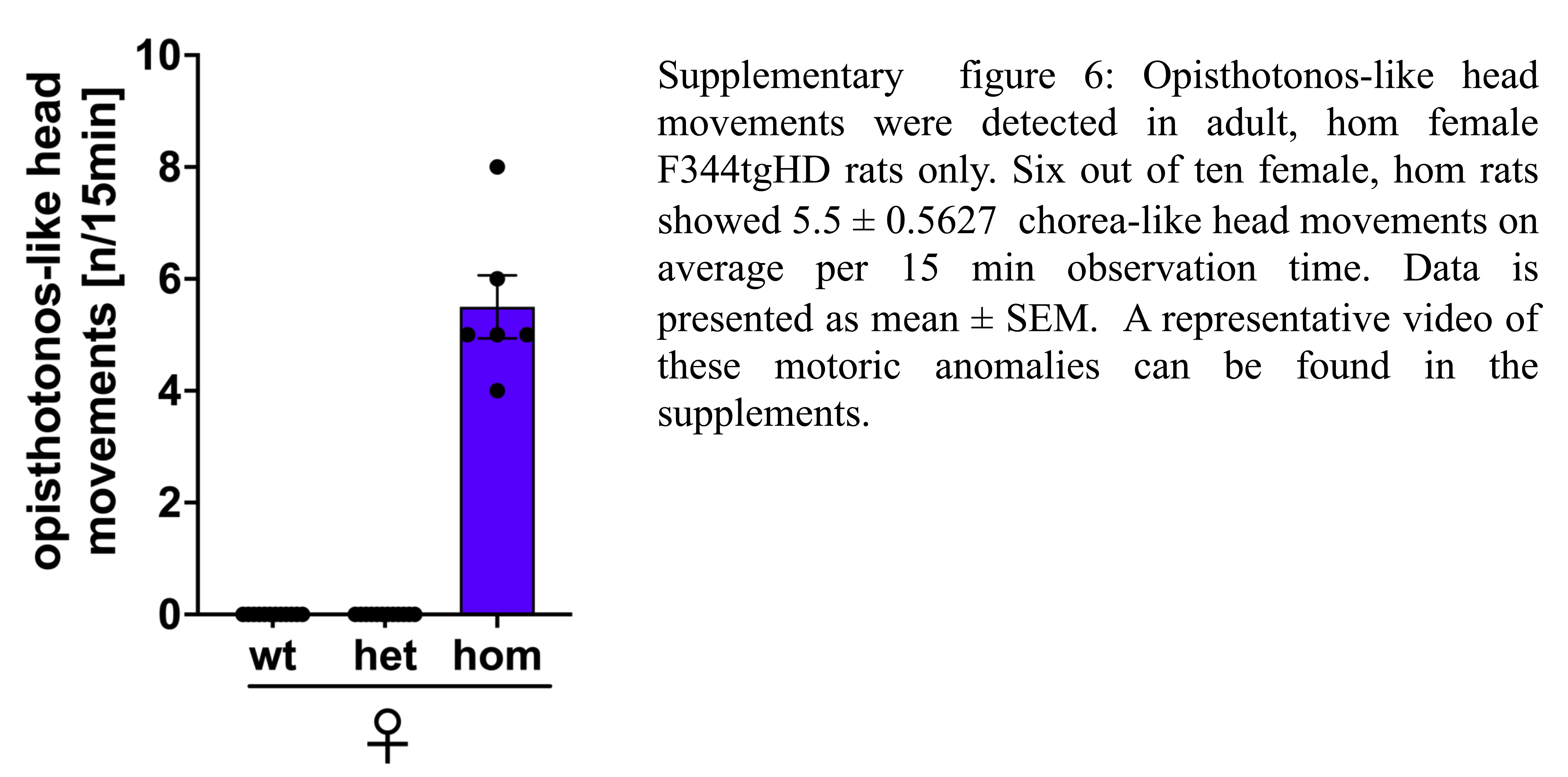

Supplement: Supplementary file 6 [file Image_6.TIFF]

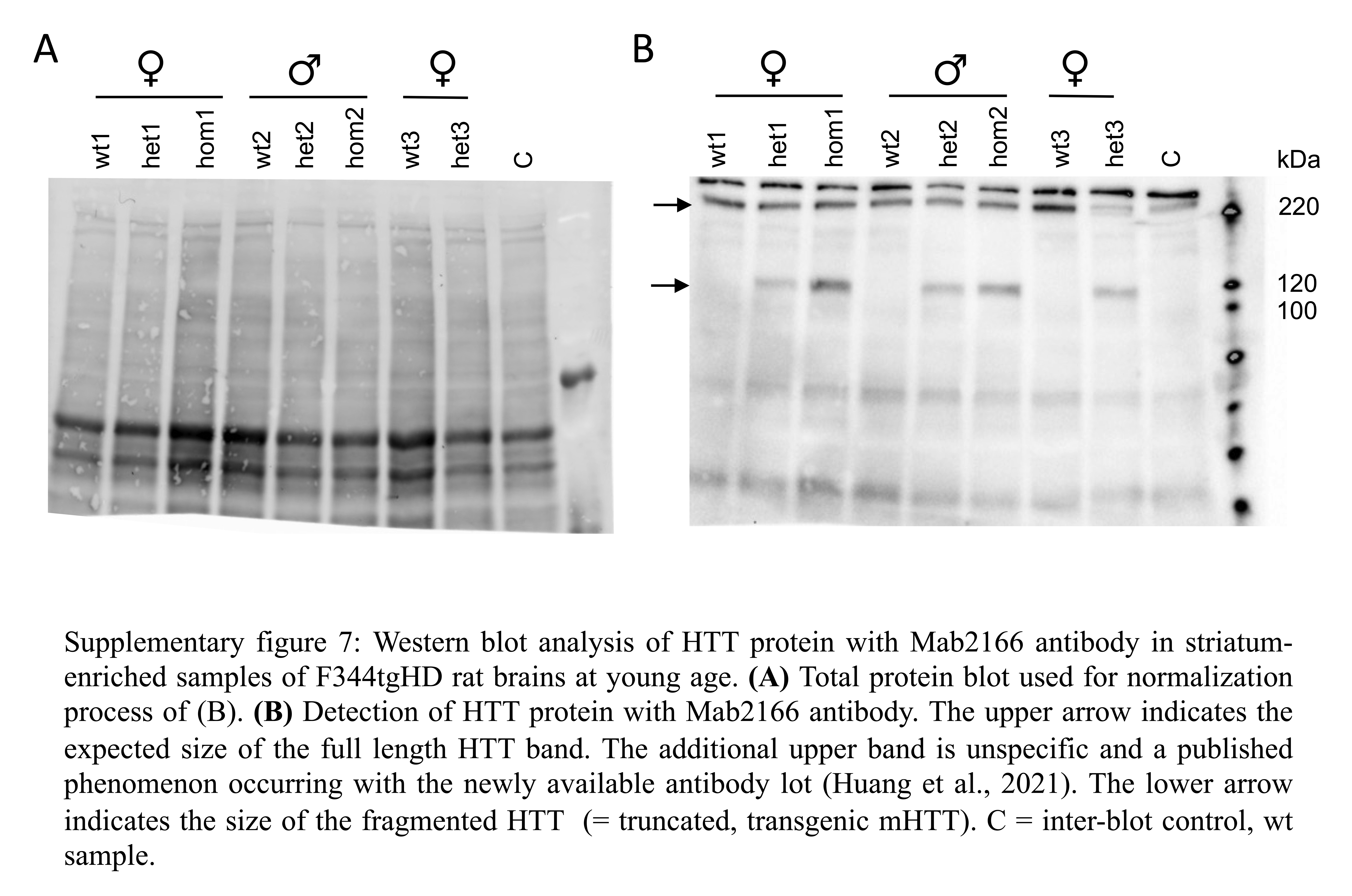

Supplement: Supplementary file 7 [file Image_7.TIFF]
