# Supplementary material for: Gene-dosage- and sex-dependent differences in the prodromal-Like phase of the F344tgHD rat model for Huntington disease
Source: Front Neurosci. 2024 Feb 7;18:1354977. doi: 10.3389/fnins.2024.1354977 (PMC10879377; doi:10.3389/fnins.2024.1354977)
Supplement: Supplementary file 8 [file Table_1.pdf]

Behavioral test	female			male		
	wt	het	hom	wt	het	hom
**ASR**	20/25/11	24/27/15	15/15/10	15/21/9	16/23/11	17/21/8
**PPI**	20/27/11	25/28/15	14/15/10	15/19/11	19/23/12	17/20/8
**Open field**	19-21/15-20/8-9	20-24/15-20/10-11	14/10-12/8-9	15-19/12-18/8	17-18/12-17/7-9	17-18/15-17/6-8
**RotaRod**	21/21/11	23/24/16	11/11/10	16/16/9	19/19/12	11/12/9
**Catwalk**	11/12/10	13/8-9/12	11/9/10	11/11-12/10	11/11-12/12	9/6-8/8
**Phenomaster**	6/12-15/6-7	11/11-12/7-8	6/5/7	5/6-9/6-9	6/9-12/5-6	3/3-12/6-8
**Social interaction**	7/9/3	5/10/3	5/4/2	5/8/2	7/7/6	5/6/3

Supplementary table 1: Number of investigated animals per behavioral test. The number of animals (n) used in each test is given in the following order: young/middle age/adult age for each group. If more than one parameter was analyzed within one test setting, the range of n is indicated. ASR: acoustic startle reaction, PPI: prepulse inhibition of ASR
